# Relationships between different types of physical activity and metabolic syndrome among Taiwanese workers

**DOI:** 10.1038/s41598-017-13872-5

**Published:** 2017-10-23

**Authors:** Jui-Hua Huang, Ren-Hau Li, Shu-Ling Huang, Hon-Ke Sia, Su-Shiang Lee, Wei-Hsun Wang, Feng-Cheng Tang

**Affiliations:** 1 Occupational Health Center, Changhua Christian Hospital, Changhua, 500 Taiwan; 2 Department of Psychology, Chung Shan Medical University, Taichung, 402 Taiwan; 3 Room of Clinical Psychology, Chung Shan Medical University Hospital, Taichung, 402 Taiwan; 4 Division of Endocrinology and Metabolism, Changhua Christian Hospital, Changhua, 500 Taiwan; 5 Department of Leisure Services Management, Chaoyang University of Technology, Taichung, 413 Taiwan; 6 Department of Orthopedic, Changhua Christian Hospital, Changhua, 500 Taiwan; 7 Department of Medical Imaging and Radiology, Shu-Zen Junior College of Medicine and Management, Kaohsiung, 821 Taiwan; 8 Department of Golden-Ager Industry Management, Chaoyang University of Technology, Taichung, 413 Taiwan; 9 Department of Occupational Medicine, Changhua Christian Hospital, Changhua, 500 Taiwan

## Abstract

This study aimed to investigate the relationships between different types of physical activity (PA) and metabolic syndrome (MetS). In this cross-sectional study, 3,296 Taiwanese workers were enrolled. A self-reported questionnaire was used to assess nutritional health behavior and PA levels related to occupation, leisure time, and commuting. Anthropometric measures, blood pressure and biochemical determinations of the blood were also obtained. Multiple logistic regression was used to evaluate the adjusted odds ratios (ORs) and 95% confidence intervals (CI) of MetS and its components associated with different types of PA. The prevalence of MetS was 16.6% in workers. Compared with a low level of leisure-time PA, a high level of leisure-time PA showed a significantly lower risk of high triglycerides (OR 0.73, 95% CI 0.61–0.87) and MetS (OR 0.76, 95% CI 0.62–0.95). Compared with a low level of occupational PA, a high level of occupational PA represented a significantly lower risk of both abdominal adiposity (OR 0.64, 95% CI 0.49–0.84) and high triglycerides (OR 0.71, 95% CI 0.55–0.90). However, commuting PA levels were not significantly associated with MetS and its components. In conclusion, occupational PA as well as leisure-time PA could be important for the prevention of MetS.

## Introduction

Metabolic syndrome (MetS) is a clustering of at least three of the five following metabolic disturbances: abdominal obesity, elevated blood pressure (BP), elevated fasting blood glucose (FBG), high triglycerides (TG), and low high-density lipoprotein cholesterol (HDL-C)^1^. MetS and its components are related to the risk of developing cardiovascular disease (CVD), diabetes, and several cancers^2–4^. The cause and risk factors for MetS include ageing, physical inactivity, and the Western diet^5,6^. Additionally, work-related factors, including sedentary work, long work periods, and high occupational stress, put workers at high risk for the development of MetS^7–9^. Accordingly, certain workers are viewed as the high-risk population with respect to the prevalence of MetS. In Taiwan, encouraging workers’ physical activity (PA) is one of the main health-promoting programs in the workplace to prevent MetS^10^.

Several findings have indicated that regular leisure-time PA would be beneficial to preventing and managing MetS and its components^11–13^. Possible mechanisms associated with regular leisure-time PA that may reduce the risk of MetS or its components include the following: (1) improving body composition through decreased abdominal adiposity^14,15^; (2) ameliorating lipid and lipoprotein profiles through lipid oxidation and then lowering TG levels, raising HDL-C levels and reducing the low-density lipoprotein cholesterol (LDL-C)-to-HDL-C ratio^16–18^; (3) ameliorating glucose homeostasis through contracting muscles to increase the uptake of blood glucose and improve insulin sensitivity^19^; (4) ameliorating BP so that the heart can pump more blood with less effort^20^. Additionally, how effectively leisure-time PA reduces the MetS risk depends somewhat on the type, intensity, duration and frequency of PA^11,21^. Compared with a low level of leisure-time PA, a high level of leisure-time PA resulted in a lower risk of MetS, but a moderate level of leisure-time PA was only weakly related to a reduced risk of MetS^11^. Therefore, engaging in at least 150 min of moderate-intensity aerobic PA weekly, at least 75 min of vigorous-intensity aerobic PA weekly, or an equivalent combination of moderate- and vigorous-intensity PA is recommended for adults to benefit their health^22^.

It has been accepted that effective PA plays an important role in the prevention of premature death and in the prevention and management of several chronic diseases^20,23,24^. In scientific research, PA includes occupational PA and commuting PA, in addition to leisure-time PA^25,26^. However, little research has been done on the effects of occupational PA and commuting PA on MetS. With respect to chronic diseases, studies showed that moderate to high levels of leisure-time PA were associated with reducing the risk of type 2 diabetes^27^, heart failure^28^, and total and CVD mortality^29^. Several studies also indicated that occupational PA might be an important factor for reducing the risk of health problems^28,30^. Moderate or high levels of occupational PA were related to reducing the risk of coronary heart disease^30^, heart failure^28^, type 2 diabetes^27^, and total and CVD mortality^29^. Similarly, commuting PA was a significant factor of a healthy lifestyle. Moderate or high commuting PA might ameliorate serum lipids^26^ and decrease the risk of type 2 diabetes^27^ and total and CVD mortality^29^. To date, worker-based studies investigating the correlations between MetS and PA have been limited. In particular, leisure-time PA, occupational PA, and commuting PA have seldom been considered simultaneously.

Among worksite-health-promotion programs, PA is one of the most common strategies for reducing the development of MetS^31^. However, discrepancies in the potential benefits of different types and levels of PA for reducing the risk of MetS or its components among workers remain and therefore require further study so that proven strategies can be defined and programs can be implemented. Therefore, we hypothesized that not only leisure-time PA but also occupational and commuting PA at an appropriate intensity may reduce the risk of MetS and its components. The objective of this study was to determine the relationships between different types and levels of PA and MetS and its components in workers.

## Methods

### Study design

The present study was conducted in 2012 by the Center for Occupational Health, using a cross-sectional research method with convenience sampling. Workers 20 years of age or older were recruited from four manufacturing companies in central Taiwan to participate in the study. Their principal activities included manufacturing of electronic components, pumps, motor vehicle parts, and transport equipment. The companies were chosen on the basis of their good relationship with the Center for Occupational Health. This allowed the study to proceed without difficulties. A total of 5,099 individuals were study candidates. Personal information, nutritional health behavior, and PA details related to leisure time, commuting, and occupation were obtained through a self-reported questionnaire. Information on each component of MetS was collected through the contacted companies’ annual health screening required by Taiwan regulations.

Under the Taiwanese Labor Health Protection Rule, occupational health personnel must routinely assess workers’ health risks and needs. This study was one component of the Taiwan Workplace-Health-Promotion Scheme and was carried out in the form of public health surveillance for workers. An information sheet attached to the questionnaire provided an explanation of the study to each candidate. The candidates were free to decide whether to take part in the study. The voluntary participants were requested to complete the questionnaire. The returned questionnaires, along with the participants’ clinical variables, were encoded by each contacted company’s occupational health personnel. Thus, on the research side, all the data collected in the study remained anonymous and were treated as strictly confidential to protect the participants’ privacy. The study was conducted according to the Declaration of Helsinki and was approved by the Institutional Review Board of the Changhua Christian Hospital in Taiwan with a waiver of informed consent (CCHIRB No: 120606).

### Assessment of nutritional health behavior

Evidence has attested to poor nutritional behavior being associated with the risk of MetS^5^; nutritional health behavior was therefore regarded as a controlling factor in this study in order to clarify the relative relationships between different types of PA and MetS. The data pertaining to nutritional health behavior were obtained using the subscale of Health-Promoting Lifestyle Profile II^32^. Nutritional health behavior included the following nine items: “choose a low-fat diet”; “limit the use of sugars”; “eat servings of bread, cereal, and rice”; “eat servings of fruit”; “eat servings of vegetables”; “eat servings of meat, poultry, fish, dried beans, eggs and nuts”; “eat servings of milk, yogurt or cheese”; “read labels to identify nutrients”; and “eat breakfast.” The number of daily servings for each food group was set according to the dietary guidelines for Taiwanese. The Taiwanese version of the subscale in this study has shown an acceptable internal consistency, with Cronbach’s alpha of 0.78. Participants were asked to rate nine items on a four-point Likert scale, ranging from 1 (never) to 4 (routinely). A higher mean score indicated a greater level of participation in nutritional health behavior.

### Assessment of different types of physical activity

A self-reported questionnaire including information about leisure-time, commuting, and occupational PA was used. The questions relating to PA were derived from those used in studies in the Nordic countries^27,33,34^. The instrument, which had been evaluated previously, had shown a high correlation with physical fitness as measured by energy expenditure^35,36^. Accordingly, the reported leisure-time PA was categorized as follows: 1) low: almost completely inactive, or doing some minor PA but not at a moderate or high level; 2) moderate: some moderate-intensity PA more than 4 h per week, e.g., walking, cycling, light gardening; and 3) high: vigorous PA more than 3 h per week, e.g., running, jogging, skiing, swimming, or heavy gardening. Daily commuting PA was categorized as follows: 1) low: using motorized transportation, or no work; 2) moderate: walking or bicycling 1–29 min; and 3) high: walking or bicycling ≧ 30 min. Occupational PA was categorized as follows: (1) low: physically very easy work with seated office work, e.g., secretarial work; (2) moderate: work including standing and walking, e.g., store assistant, light industrial work; and (3) high: work including walking and lifting, or heavy manual labor, e.g., industrial work, farm work^27,33^.

### Components of metabolic syndrome

Components of MetS, including waist circumference (WC), BP, FBG, TG, and HDL-C, were collected through employee health screening reports. MetS was defined as the presence of three or more of the following risk determinants: (1) increased waist circumference (Health Promotion Administration, Taiwan, has categorized WC ≧ 90 cm for men or ≧ 80 cm for women as abdominal adiposity); (2) high BP [systolic BP (SBP) ≧130/diastolic BP (DBP) ≧ 85 mmHg]; (3) impaired FBG (≧100 mg/dl); (4) elevated TG (≧150 mg/dl); and (5) decreased HDL-C (<40 mg/dl in men, <50 mg/dl in women)^1,37^.

The measurement of WC (cm) was conducted by trained health personnel according to International Standards for Anthropometry and Kinesiology (ISAK)^38^. It was measured at the narrowest waist level, or if this was not apparent, at the midpoint between the lowest rib and the top of the hip bone (iliac crest) using a measuring tape. Measurement was made at the end of a normal expiration. Before reading the tape measure, it was ensured that the tape was snug but did not compress the skin and was parallel to floor. In measuring BP, the BP reading was taken once from the upper arm using a validated digital sphygmomanometer (HEM-7310, Omron, Kyoto, Japan) after the participant was seated for at least five minutes. The procedure was carried out based on the practice guidelines recommended by the European Society of Hypertension^39^. The common factors affecting the accuracy, including selecting an appropriate device, adequately explaining to the participant, the posture and the attitude of the participant, the attitude of the observer, and so on, were considered during the measurement.

Blood samples were obtained with subjects in the fasting status for at least 8 hours, and biochemical measurements of blood were performed by the medical laboratory (certified ISO 15189). Fasting plasma glucose and serum lipids, including TG and HDL-C, were assayed using a biochemical auto-analyzer (TBA-200FR, Toshiba, Tokyo, Japan). FBG was measured by an Enzymatic UV test (hexokinase method). TG was assessed using a series of coupled enzymatic reactions. HDL-C values were measured by direct methods. The intra- and inter- assay coefficients of variation were 0.8% and 2.2%, respectively, for FBG, 0.6% and 2.1% for TG, and 0.8% and 3.5% for HDL-C.

### Statistical Analysis

Data were analyzed by the 2-tailed t-test (2 groups) and one-way ANOVA (å 2 groups) for continuous variables and frequency distribution for categorical variables. Relationships between leisure-time, commuting, and occupational PA and components of MetS were examined simultaneously by multiple logistic regression analysis with adjustment for gender, age, and nutritional health behavior. The odds ratios (ORs) and their 95% confidence intervals (95% CI) were calculated for each potential risk factor. All statistical procedures were performed using SPSS (version 17.0) statistical software (SPSS Inc., Chicago, IL, USA), and a p-value of 0.05 was considered statistically significant.

## Results

A total of 4,154 workers, 20 years of age or more, volunteered for the current study. However, some of the workers did not provide the necessary information on personal data, daily PA, nutritional health behavior, or a physical examination, resulting in a final number of 3,296 participants, with an average age of 43.0 ± 9.9. Using their information, characteristics pertaining to PA levels, nutritional health behavior, and MetS and its parameters were summarized in Table 1. The male workers had significantly higher rates of high leisure-time PA (p < 0.001), high commuting PA (p = 0.005), and high occupational PA (p < 0.001) than the female workers. Compared with the male workers, the female workers had significantly higher nutritional health behavior scores and HDL-C and significantly lower fasting plasma glucose, TG, SBP, DBP and WC (p < 0.001). In addition, the male workers had a significantly higher rate of MetS.

**Table 1:** Baseline characteristics of the workers^1,2^.

Variables^3^	Total N = 3296	Male n = 2696	Female n = 600	p
Age (y)	43.0 ± 9.9	42.4 ± 19.3	45.5 ± 7.5	<0.001
Nutritional health behavior score	2.47 ± 0.43	2.45 ± 0.43	2.59 ± 0.45	<0.001
Leisure-time PA				
Low	1374 (41.7)	1082 (40.1)	292(48.7)	<0.001
Moderate	623 (18.9)	497 (18.4)	126 (20.0)	
High	1299 (39.4)	1117 (41.4)	182 (30.3)	
Commuting PA				
Low	733 (22.2)	579 (21.5)	154 (25.7)	0.005
Moderate	1913 (58.0)	1560 (57.9)	355 (58.8)	
High	650 (19.7)	557 (20.7)	93 (15.4)	
Occupational PA				
Low	1297 (39.4)	863 (32.0)	436 (72.3)	<0.001
Moderate	1390 (42.1)	1274 (47.3)	116 (19.2)	
High	610 (18.5)	559 (20.7)	51 (8.5)	
Metabolic parameters				
WC (cm)	82.0 ± 9.4	83.9 ± 8.7	73.4 ± 8.1	<0.001
FBG (mg/dL)	94.0 ± 19.6	94.3 ± 19.6	89.6 ± 14.4	<0.001
SBP (mmHg)	123.2 ± 15.4	124.4 ± 14.8	116.5 ± 16.8	<0.001
DBP (mmHg)	78.7 ± 11.2	79.1 ± 11.3	76.8 ± 11.0	<0.001
TG (mg/dL)	134.6 ± 99.4	139.2 ± 100.7	110.3 ± 78.3	<0.001
HDL-C (mg/dL)	52.8 ± 12.6	50.7 ± 11.5	61.0 ± 13.0	<0.001
Metabolic syndrome				
<3 components	2849 (83.4)	2306 (85.5)	543 (90.5)	0.001
≧3 components	447 (16.6)	390 (14.5)	57 (9.5)	

^1^Continuous variables were presented as mean values and standard deviation (Mean ± SD).

^2^Categorical variables were analyzed according to frequency distribution and expressed in number and percentages [n (%)].

^3^Abbreviations: PA, Physical activity; WC, Waist circumference, FBG, Fasting blood glucose; SBP, Systolic blood pressure; DBP, Diastolic blood pressure; TG, Triglyceride; HDL-C, High-density lipoprotein cholesterol.

As shown in Table 2, the workers with a high level of leisure-time PA had significantly higher HDL-C (p = 0.032) and trended to a lower WC (p = 0.053) than those with a low or moderate level of leisure-time PA. The workers with a high level of occupational PA had a significantly lower WC (p = 0.001) and higher SBP (p = 0.013) and HDL-C (p = 0.033) than those with a low or moderate level of occupational PA. Furthermore, the workers with a high level of occupational PA exhibited a significantly lower DBP (p = 0.001) and TG (p = 0.011) than those with a moderate or low level of occupational PA. However, commuting PA was not significantly associated with metabolic parameters.

**Table 2:** Relationship of different physical activity types and metabolic parameters^1^.

Variables^2^	WC (cm)	FBG (mg/dL)	SBP (mm Hg)	DBP (mm Hg)	TG (mg/dL)	HDL-C (mg/dL)
**Physical activity types**						
Leisure-time PA						
Low	79.0 ± 0.3	92.2 ± 0.6	119.5 ± 0.4	77.4 ± 0.3	127.1 ± 2.9	55.4 ± 0.4
Moderate	78.3 ± 0.4	92.0 ± 0.8	120.1 ± 0.6	77.7 ± 0.4	124.4 ± 4.1	55.2 ± 0.5
High	78.1 ± 0.3^§^	91.3 ± 0.6	120.7 ± 0.5	77.8 ± 0.3	120.7 ± 3.2	56.5 ± 0.4^§, ||^
*p*	0.053	0.464	0.160	0.606	0.257	0.032
Commuting PA						
Low	78.4 ± 0.3	92.1 ± 0.7	119.5 ± 0.6	77.6 ± 0.4	125.4 ± 3.8	55.8 ± 0.5
Moderate	78.5 ± 0.2	91.5 ± 0.5	120.0 ± 0.4	77.5 ± 0.3	124.2 ± 2.6	55.5 ± 0.3
High	79.1 ± 0.4	92.4 ± 0.8	121.1 ± 0.6	77.9 ± 0.5	123.8 ± 4.2	56.4 ± 0.5
*p*	0.272	0.552	0.135	0.710	0.945	0.287
Occupational PA						
Low	78.8 ± 0.3	91.4 ± 0.6	119.4 ± 0.4	78.4 ± 0.3^‡,§^	130.1 ± 2.9^‡,§^	55.2 ± 0.4
Moderate	78.7 ± 0.3	92.3 ± 0.6	120.1 ± 0.5	76.8 ± 0.3	120.1 ± 3.2	55.9 ± 0.4
High	77.3 ± 0.4^§, ||^	92.1 ± 0.9	121.7 ± 0.7^§, ||^	76.6 ± 0.5	116.0 ± 4.5	56.9 ± 0.5^§^
*p*	0.001	0.573	0.013	0.001	0.011	0.033

^1^Relationships of different physical activity types and metabolic parameters were examined by one way ANOVA tests followed by LSD post-hoc test.

^‡^indicates significant differences between low and moderate physical activity.

^§^indicates significant differences between low and high physical activity.

^||^indicates significant differences between moderate and high physical activity.

^2^Abbreviations: PA, Physical activity; WC, Waist circumference, FBG, Fasting blood glucose; SBP, Systolic blood pressure; DBP, Diastolic blood pressure; TG, Triglyceride; HDL-C, High-density lipoprotein cholesterol.

Table 3 shows the adjusted ORs of MetS and its components according to PA. After adjustments for gender, age, and nutritional health behavior, a high level of leisure-time PA showed a significantly lower risk of high TG (OR 0.73, 95% CI 0.61 to 0.87) and MetS (OR 0.76, 95% CI 0.62 to 0.95) than did a low level of leisure-time PA. Compared with a low level of occupational PA, a high level of occupational PA showed a significantly lower risk of abdominal adiposity (OR 0.64, 95% CI 0.49 to 0.84). Moreover, the ORs of high TG for a high and moderate level of occupational PA were 0.71 and 0.75, respectively. These two ORs were statistically significant. However, the commuting PA levels were not significantly associated with the components of MetS. In addition to the main findings, nutritional health behavior itself was found to be negatively associated with the risk of high TG and MetS.

**Table 3:** Odds ratio of metabolic syndrome and its components by the type of physical activity^1^.

Variables^2^	WC (cm) ≧90 for men or ≧80 for women	p	FBG (mg/dL) ≧100	p	Blood pressure SBP (mm Hg) ≧130/DBP (mm Hg) ≧85	p	TG (mg/dL) ≧150	p	HDL-C (mg/dL) <40 for men or <50 for women	p	Metabolic Syndrome	
**Age (years)**	1.01 (1.00–1.02)	0.018	1.03 (1.02–1.04)	<0.001	1.05 (1.05–1.06)	<0.001	1.02 (1.02–1.03)	<0.001	0.98 (0.97–0.99)	<0.001	1.03 (1.02–1.04)	<0.001
**Gender**												
Female	referent		referent		referent		referent		referent		referent	
Male	1.27 (1.01–1.61)	0.043	2.26 (1.68–3.04)	<0.001	2.48 (2.01–3.06)	<0.001	2.81 (2.20–3.61)	<0.001	0.60 (0.46–0.77)	<0.001	1.90 (1.43–2.54)	<0.001
**Nutritional health behavior**.	0.82 (0.66–1.01)	0.063	0.94 (0.75–1.18)	0.604	0.92 (0.77–1.11)	0.395	0.80 (0.66–0.98)	0.028	1.01 (0.79–1.28)	0.953	0.67 (0.53–0.85)	0.001
**Physical activity types**												
leisure-time PA												
Low	referent		referent		referent		referent		referent		referent	
Moderate	0.95 (0.76–1.20)	0.679	0.91 (0.71–1.18)	0.487	0.94 (0.77–1.15)	0.542	0.81 (0.65–1.01)	0.058	1.11 (0.86–1.43)	0.441	0.93 (0.72–1.20)	0.559
High	0.91 (0.75–1.10)	0.315	0.89 (0.72–1.10)	0.271	1.03 (0.87–1.22)	0.941	0.73 (0.61–0.87)	0.001	0.84 (0.67–1.05)	0.128	0.76 (0.62–0.95)	0.014
Commuting PA												
Low	referent		referent		referent		referent		referent		referent	
Moderate	1.00 (0.81–1.23)	0.989	1.03 (0.82–1.29)	0.799	1.03 (0.86–1.24)	0.754	0.99 (0.81–1.20)	0.880	1.05 (0.83–1.33)	0.688	1.11 (0.88–1.40)	0.395
High	1.23 (0.95–1.59)	0.114	0.99 (0.75–1.31)	0.941	1.09 (0.87–1.36)	0.479	0.93 (0.72–1.19)	0.542	1.01 (0.74–1.37)	0.970	1.06 (0.79–1.42)	0.694
Occupational PA												
Low	referent		referent		referent		referent		referent		referent	
Moderate	0.89 (0.74–1.09)	0.266	1.17 (0.94–1.45)	0.166	0.88 (0.74–1.05)	0.166	0.75 (0.63–0.91)	0.003	0.99 (0.78–1.25)	0.926	0.89 (0.72–1.11)	0.288
High	0.64 (0.49–0.84)	0.001	1.18 (0.89–1.55)	0.249	0.99 (0.80–1.24)	0.956	0.71 (0.55–0.90)	0.005	1.00 (0.75–1.34)	0.990	0.84 (0.63–1.12)	0.095

^1^Physical activity types were analyzed simultaneously by multiple logistic regression analysis with adjustment for gender, age and nutritional health behavior. Data are odds ratios (95% CIs).

^2^Abbreviations: PA, Physical activity; WC, Waist circumference, FBG, Fasting blood glucose; SBP, Systolic blood pressure; DBP, Diastolic blood pressure; TG, Triglyceride; HDL-C, High-density lipoprotein cholesterol.

## Discussion

Our investigation addressed the associations between different types of PA and the risk of MetS in workers. The types of PA included leisure-time PA, occupational PA, and commuting PA. A high level of leisure-time PA represented a lower WC, a higher HDL-C, and a lower risk of high TG and MetS. A high level of occupational PA also represented a lower WC, a higher HDL-C, and a higher SBP and lower DBP. In addition, a moderate or high level of occupational PA represented a lower risk of high TG. Nevertheless, commuting PA was not significantly associated with MetS and its components.

Our data indicated that workers with a high level of leisure-time PA showed a lower WC and a higher HDL-C. Moreover, a high level of leisure-time PA was associated with a lower risk of high TG and MetS after adjustment for commuting, occupational PA, and other known impact factors. A previous study reported that vigorous exercise was related to no weight gain even in sedentary occupations^40^. A meta-analysis of controlled trials reported after dynamic endurance training showed a decrease in WC, BP, and LDL-C and an increase in HDL-C, but such training had no impact on TG^41^. Amelioration in body composition such as decrement in abdominal adiposity or WC characteristics was suggested to be associated with beneficial alterations in lipid and lipoprotein profiles through mechanisms related to lipid oxidation and insulin resistance^42–45^. These effects also may further benefit reducing the risk of MetS. Indeed, our data were in line with several studies showing that a higher level of leisure-time PA was related to a lower risk of MetS^11–13^. Lifestyle interventions, including appropriate leisure-time PA, may improve metabolic abnormalities and are highly effective in preventing or delaying the onset of MetS and its components^46^. Therefore, having workers maintain a regular and appropriate leisure-time PA could be one of the goals of workplace health promotion.

Few studies pertained to the effects of occupational PA on preventing the development of MetS and its components. Relevant studies mainly focused on relationships between occupational PA and cardiovascular disease or type 2 diabetes. Hu *et al*. reported that moderate or high levels of occupational PA were related to a lower risk of coronary heart disease, heart failure, type 2 diabetes, and total and CVD mortality^27–30^. A study showed that non-manual work correlated with MetS, a high body mass index, and insulin resistance^8^. One of our main findings was that workers with a high level of occupational PA had a lower WC and higher HDL-C; there was also an association with a lower DBP. Moreover, workers with a moderate or high level of occupational PA had a lower risk of high TG after adjustment for leisure-time, commuting PA, and other known impact factors. These findings suggested that occupational PA might also be a considerable factor for the development of MetS among workers. Increased computerization and mechanization as well as convenient transportation lead to an increasingly sedentary lifestyle, which tends to reduce workers’ daily PA. Therefore, workers doing jobs with a low level of occupational PA should be more concerned about their PA and the potential development of MetS. Our results suggested that a moderate or high level of occupational PA was significantly negatively associated with some components of MetS. Nevertheless, we also found that workers with a high level of occupational PA had a higher SBP. Further investigation is necessary to determine whether this finding resulted from occupational stress or other factors.

Daily commuting PA is a component of an individual’s total PA. Several studies indicated that a moderate or high level of commuting PA may ameliorate serum lipids and decrease the risk of type 2 diabetes and total and CVD mortality^26,27,29^. Other studies also suggested that daily walking or cycling to and from work was associated with reducing MetS components and raising HDL-C^26,47,48^. However, significant relationships between MetS and daily commuting PA were not found in the present study. A possible reason for this is that the effects of daily commuting PA on MetS were not that great when leisure-time PA and occupational PA were considered together. Further study to explore whether the impact of commuting PA on MetS components has potential utility is recommended.

Insufficient PA has been found to have a negative impact on physical work capacity and health^49,50^. Appropriate PA is an essential part of workplace health-promotion strategies for improving health and preventing MetS and its components^50^. It is important for decision makers in workplace health promotion to understand these risks and benefits so that they are able to make informed recommendations to workers about how appropriate PA has health-related benefits. The present study’s findings could have some policy implications for promoting good health in the workplace. Workers with a high level of leisure-time PA had lower odds ratios of high TG and MetS. However, the fact that the percentage of a low leisure-time PA level in all study participants was more than 40% shows that there is clearly still room for improvement. The development of more-effective strategies for helping workers adhere to good leisure-time PA habits is recommended. Given that a moderate to high level of occupational PA had lower odds ratios of abdominal adiposity and high TG, workplace health-promotion practitioners should consider the occupational PA levels of workers when designing their health-promoting exercise programs aimed at reducing MetS.

Many factors may increase the risk of MetS and its components. Among these, physical inactivity and poor nutritional behavior have been evidenced^5,6^. Although nutritional health behavior was regarded as a covariate in the present study, poor nutritional health behavior did show significantly associations with the occurrence of MetS and high TG. To promote employee health, nutritional health should be encouraged. There was a discrepancy shown in the potential benefits of different types and levels of PA for reducing the risk of MetS and its components. This study provided insights into the relationships between different PA types and MetS for promoting health in the workplace; nevertheless, it had several limitations. First, it was a cross-sectional study and could not determine the causal conjunction between different PA types and MetS and its components. Second, the analyses of various levels of PA were highly dependent on data collected from a self-reported questionnaire. Overestimation or underestimation may have occurred when participants described their own habitual PA. To overcome these drawbacks, more longitudinal studies with objective tools for assessing PA are recommended. Lastly, due to the limit of the fast-paced employee health screening at the workplace in the present study, taking a series of BP readings and WC measurements was not applicable. In regard to the variability of BP and potential errors in the measurement of WC, additional measurements in future study are strongly advised.

## Conclusion

This study suggests that a high level of leisure-time PA is related to a lower risk of MetS. We also find that occupational PA shows significant associations with some components of MetS. The randomized controlled trial would be the next phase to test whether leisure time PA and occupational PA will be assist in reducing MetS. Since low leisure-time PA and low occupational PA were both around 40% in all the participants, it is recommended that leisure-time PA and occupational PA be considered as factors in an essential focus on workplace health promotion.
